# The effect of environmental factors on technical and scale efficiency of primary health care providers in Greece

**DOI:** 10.1186/1478-7547-5-14

**Published:** 2007-11-17

**Authors:** Nick Kontodimopoulos, Giorgos Moschovakis, Vassilis H Aletras, Dimitris Niakas

**Affiliations:** 1 Hellenic Open University, Faculty of Social Sciences, Riga Feraiou 169 & Tsamadou, 26222, Patras, Greece; 2 University of Macedonia, Department of Business Administration, Egnatia 156, P.O. Box 1591, 54006, Thessaloniki, Greece

## Abstract

**Background:**

The purpose of this study was to compare technical and scale efficiency of primary care centers from the two largest Greek providers, the National Health System (NHS) and the Social Security Foundation (IKA) and to determine if, and how, efficiency is affected by various exogenous factors such as catchment population and location.

**Methods:**

The sample comprised of 194 units (103 NHS and 91 IKA). Efficiency was measured with Data Envelopment Analysis (DEA) using three inputs, -medical staff, nursing/paramedical staff, administrative/other staff- and two outputs, which were the aggregated numbers of scheduled/emergency patient visits and imaging/laboratory diagnostic tests. Facilities were categorized as small, medium and large (<15,000, 15,000–30,000 and >30,000 respectively) to reflect catchment population and as urban/semi-urban or remote/island to reflect location. In a second stage analysis, technical and scale efficiency scores were regressed against facility type (NHS or IKA), size and location using multivariate Tobit regression.

**Results:**

Regarding technical efficiency, IKA performed better than the NHS (84.9% vs. 70.1%, Mann-Whitney *P *< 0.001), smaller units better than medium-sized and larger ones (84.2% vs. 72.4% vs. 74.3%, Kruskal-Wallis *P *< 0.01) and remote/island units better than urban centers (81.1% vs. 75.7%, Mann-Whitney *P *= 0.103). As for scale efficiency, IKA again outperformed the NHS (89.7% vs. 85.9%, Mann-Whitney *P *= 0.080), but results were reversed in respect to facility size and location. Specifically, larger units performed better (96.3% vs. 90.9% vs. 75.9%, Kruskal-Wallis *P *< 0.001), and urban units showed higher scale efficiency than remote ones (91.9% vs. 75.3%, Mann-Whitney *P *< 0.001). Interestingly 75% of facilities appeared to be functioning under increasing returns to scale. Within-group comparisons revealed significant efficiency differences between the two primary care providers. Tobit regression models showed that facility type, size and location were significant explanatory variables of technical and scale efficiency.

**Conclusion:**

Variations appeared to exist in the productive performance of the NHS and IKA as the two main primary care providers in Greece. These variations reflect differences in primary care organization, economical incentives, financial constraints, sociodemographic and local peculiarities. In all technical efficiency comparisons, IKA facilities appeared to outperform NHS ones irrespective of facility size or location. In respect to scale efficiency, the results were to some extent inconclusive and observed differences were mostly insignificant, although again IKA appeared to perform better.

## Background

### Primary care in Greece

In Greece, primary care is provided mostly through the National Health System (NHS) with approximately 200 rural and semi-urban primary care centers and 1,000 rural health posts, and the Social Security Foundation (IKA) with approximately 250 urban facilities of its own. Other primary care providers are the outpatient departments of NHS public hospitals for the urban population, other insurance funds, the private sector, local authorities and city health departments. Interestingly, improvement of primary care has been in the agenda of health reform attempts in Greece over the past 25 years.

Planning and staffing of NHS primary care centers is based on a combination of population-, demographic-, economic- and geographic-based criteria. These units provide preventive, curative and rehabilitation services to people living in their service areas, and to visitors as well. They are staffed with salaried GPs and internists, nurses, lab technologists and assistants and other health and administrative personnel. Rural posts are staffed with one physician, usually a GP. NHS primary care centers are mainly tax-financed and their budgets are linked to staff numbers and other inputs.

These NHS centers have partially fulfilled their objectives by increasing access to primary care and reducing the flow of rural patients to urban hospital outpatient departments. However, due to staffing, financial and organizational problems, they have been yet unable to develop their own policies and their performance has not attained expected standards [1-3]. Moreover, they are in a monopolistic position, particularly in remote areas and islands, and this weakens cost minimization and performance improvement incentives.

IKA, on the other hand, is the largest public health insurance entity and the main public-sector provider of primary health care services, covering more than half of the insured population. It operates its own network of contracted doctors and primary care centers to which its patients have free access. IKA's health care provision is financed primarily by contributions from employees and their employers. Facilities are staffed with part-time salaried physicians covering almost all medical specialties (who concurrently maintain private practice), nurses and other health personnel.

### Technical and scale efficiency

Technical efficiency depicts the capability of production units to transform their inputs into outputs. In this sense, a primary care center is perceived as efficient if it produces the maximum possible output, given its available inputs or, equivalently, if it utilizes a minimum level of inputs to produce a given amount of outputs. As the ideal "maximum" or "minimum" level is unknown, efficiency is practically measured in comparative terms to other units [4]. Scale efficiency can be assessed in terms of production by referring to the notion of returns to scale. Increasing returns are said to exist when a proportional increase in inputs causes outputs to increase by a greater proportion, whereas decreasing returns is the situation in which an increase in inputs causes output to increase by a smaller proportion.

### Data Envelopment Analysis

Data Envelopment Analysis (DEA) is a linear programming technique for identifying optimal combinations of inputs and outputs based on the actual performance of comparable units, and a unit-by-unit empirical frontier is created representing, in economic terms, the revealed best practice production technology. The efficiency of a production unit not located on the frontier is estimated by comparing its performance with efficient units with the most similar production characteristics. The first DEA model made the dubious assumption of constant returns to scale (CRS) [5], but was subsequently developed to measure technical efficiency under variable returns to scale (VRS) [6,7].

In DEA, a linear programming formulation is solved for each unit in order to maximise efficiency, which is defined as the weighted sum of outputs to the weighted sum of inputs. Weights are chosen to show the specific unit in the most positive light as possible, under the restriction that no other unit, given the same weights, is more than 100% efficient. The underlying concept is that a benchmark, which is a convex combination of efficient units, can virtually exist and inefficient ones should attempt to emulate its practices to become themselves efficient. The input-oriented model focuses on the minimization of inputs and calculates the degree to which each production unit can radially reduce the quantities of utilized inputs in order to still produce a given amount of outputs. In contrast, the output-oriented model calculates efficiency as the percentage increase in outputs that is feasible by a given available quantity of inputs.

DEA has a number of strengths such as being underpinned by economic theory and methods, focusing on relative (not absolute) efficiency, ability to incorporate multiple inputs and outputs simultaneously and identifying actual good practice and performance targets. As a non-parametric technique, it does not require knowledge of the underlying production function as opposed to typical econometric models. However, in practice it can be challenging to characterize the production process validly, particularly when an unmanageable number of variables may be required to capture it adequately or the quality of available data may be too poor to provide accurate measurement and produce valid results. As a data-driven deterministic technique, results are highly sensitive to outlier observations, insensitive to statistical noise and the measurement of comparative efficiency rests on the hypothesis that efficient units are genuinely efficient.

Since its introduction, DEA has been improved and adopted in a variety of uses in for-profit and not-for-profit situations [8] and has been validated by observations, simulations and hypothetical data sets with known efficiencies [9]. Applications of DEA in health care management have raised important questions on individual performance of production units. The literature contains extensive reviews of studies in which parametric and mostly non-parametric methods have been employed to assess the productive performance of hospitals and health care services [10,11]. DEA has also been used to study efficiency in the context of primary health care centers [12-15].

Standard DEA models incorporate only inputs, which are controllable at the unit level. However, socio-economic, environmental and other exogenous factors, known as non-discretionary inputs, may be important in determining efficiency variations across facilities, particularly in public sector production applications [16]. The effect of such factors is usually explored with "second stage DEA", for which various approaches have been described [17]. The most often encountered approach is the two-limit Tobit technique, which has been adopted as the natural choice for modelling DEA scores in second stage evaluations. This method is suitable when the dependent variables are censored or corner solution outcomes [18], as in the case of DEA scores which are continuous on the [0–1] interval and take the value 1 with positive probability, while the probability of obtaining the limiting-value 0 is zero. Tobit regression has been used in various efficiency studies in health care, such as in hospitals [19-21], nursing homes [22,23], oral health provision [24] and for estimation of physician efficiency [25].

### Efficiency measurement of Greek primary care

An overview of the fairly limited efficiency studies involving the Greek health care sector, using non-parametric and parametric methods, has been provided elsewhere [26,27]. A common finding in most of these studies was the potential for considerable improvement regarding both technical and scale efficiency and those specifically involving primary care did not differ in this respect. An early study evaluated the operation of primary health centers in terms of inputs and outputs and the extent to which organization of primary health in Greece was in coherence to WHO guidelines [28]. Another Greek study involving 133 IKA primary care centers showed that those with eligible covered populations from 10,000 to 50,000 were the most efficient and suggested that population size and vulnerability should be considered in resource allocation and policymaking [29]. One study examined the efficiency of 24 NHS centers in rural and semi-urban areas and concluded that those located close to secondary or tertiary hospitals suffered from higher inefficiencies [30]. Recent evidence from small-scaled hospitals-known as hospital/health centers-located on small islands or in remote mainland areas in Greece and serving populations less than 20,000, showed average technical inefficiencies of about 25% and raised the concern that efforts to improve efficiency could compromise access equity for the respective populations [31].

The present study is the first to jointly involve primary care facilities from the NHS and IKA and to attempt to interpret potential efficiency variations based on their structural and organizational differences. Furthermore, it specifically focuses on the issues of catchment population (i.e. the area and population from which the primary care facilities attract patients) and location (urban/semi-urban and remote/island) and on the extent to which these affect technical and scale efficiency. Primary care centers were classified as small, medium and large if their service populations were <15,000, 15,000–30,000 and >30,000 respectively.

## Methods

### Data collection

The NHS Regional Health Systems, which oversee the operation of primary care centers in their respective areas, and the central administration of IKA for its own facilities, provided all the data for this study. These data included staff numbers according to specialty, i.e. physicians, nurses, paramedical, administrative and other support staff, numbers of scheduled and emergency (the latter applicable only to NHS) patient visits and numbers of performed laboratory and radiographic examinations. Data collection was performed under the responsibility of the Hellenic Open University. Initially, raw 2004 data for 133 out of 196 NHS (response rate 67.9%) and 118 out of 204 IKA facilities (response rate 57.8%) was collected and examined for completeness and clarity. In order to form a reasonably homogenous set of health centers, extremely large (>300 staff) and small (<8 staff) units were excluded from the sample, since the former emulate practices of small hospitals, while the latter of rural posts. Centers reporting zero inputs or outputs for any of the variables and those suspected of reporting unreliable data (i.e. unusually large or small amounts of outputs) were also removed from the sample. These procedures resulted in the final sample consisting of 103 NHS and 91 IKA primary care centers.

### DEA model specification

Input-output selection in DEA is usually guided by expert opinion, past experience and economic theory and there are no tests for model misspecification, which is most serious when relevant variables are omitted rather than when irrelevant ones are included [32]. Moreover, difficulties related to the measurement of outputs in healthcare facilities are well known and refer mainly to the nature and variety of functions [33]. It is hence typical to employ intermediate outputs/services in hospital cost or production analyses (e.g. patient days, number of cases, etc.). Furthermore, delivery of primary health care is diverse and there is little consensus on a common operational definition or on the identification of inputs, outputs and processes [34].

In this study, two outputs were selected to reflect production responsibilities of primary care centers. Specifically, aggregated scheduled and emergency outpatient visits and aggregated laboratory and radiographic tests performed. Inputs were three categories of staff as the main providers of services, namely physicians, nursing/paramedical and administrative/support staff. Centers were assumed to have no control over the number of patients treated and, as a result, limited control over the number of examinations performed. It is more logical to assume that they can control utilization of resources, implying that an input-oriented DEA model should be adopted. A *benchmarking *approach, in which efficient DMUs are measured in terms of their importance as benchmarks for the inefficient ones [35], was used in this study. The DEA software *Efficiency Measurement System *[36] was used to compute efficiency scores and to identify best-practice units.

### Second stage analysis

In the second part of the study, the estimated technical and scale efficiency scores were regressed against a set of environmental characteristics, which reflect differences in primary care organization, economic incentives, financial constraints, geographic and demographic factors and local peculiarities. Three explanatory variables, all beyond the influence of managerial control, were chosen to be included in a Tobit regression: i) catchment population (small, medium and large) to account for the scale of operation and differences in input mix, ii) provider (NHS vs. IKA) to account for structural and organizational differences in health care provision and iii) location (urban/semi-urban vs. remote/island) to account for accessibility and population demographics. These analyses were performed with STATA ver. 8.0.

## Results

Mean inputs and outputs for the 103 NHS and 91 IKA primary care centers according to size-proxied by the respective catchment populations – are presented in Table 1. IKA centers treated more outpatients and performed more diagnostic examinations compared to their NHS counterparts, and this was even more obvious in medium- and large-sized facilities in which outputs were four to six times higher. Mean catchment population was approximately 50% larger for large-sized IKA centers, compared to the respective NHS ones, and this was the result of IKA centers being located primarily in urban areas. Concerning inputs, IKA employed more physicians and nursing/paramedical staff, whereas the NHS more administrative and other support staff. This might have been expected since NHS centers are located mostly in rural and remote areas or on islands, implying difficulties in attracting specialized personnel.

**Table 1:** Mean inputs and outputs of NHS (N = 103) and IKA (N = 91) primary care centers by size^1^

			**Inputs**			**Outputs**	
				
**NHS**	N	Mean catchment population	Physicians	Nursing/Paramedical staff	Administrative/Other staff	Outpatients	Diagnostic Tests
		
Small	36	9325	8.19	7.75	6.61	26732	24536
Medium	35	21722	10.94	13.71	9.60	32523	41305
Large	32	43218	16.16	23.09	11.66	49672	61473

**IKA**							
							
Small	30	9759	18.00	6.10	1.93	56335	31384
Medium	25	23218	37.56	20.40	3.48	126149	146302
Large	36	67102	93.97	36.14	6.50	290674	308833

^1 ^Size according to catchment population, i.e. small (<15,000), medium (15,000–30,000) and large (>30,000)

Table 2 shows DEA results using the entire sample. CRS and VRS efficiency were 67.3% and 77.1% respectively (Wilcoxon test, *P *< 0.001), with the observed difference implying the existence of scale inefficiencies as well, which were 12.3% on average. The first direct comparison between primary care providers revealed that IKA centers were on average 14.8% more technically efficient than their NHS counterparts (Mann-Whitney, *P *< 0.001) and 3.9% more scale efficient as well. Catchment population evidently affected VRS efficiency and specifically smaller facilities were the most efficient (Kruskal-Wallis, *P *= 0.002). Medium and large primary care centers were comparable, demonstrating overall technical inefficiencies between 25.7–27.6%. The opposite was observed regarding scale efficiency, since larger centers achieved significantly higher scores. Centers were classified according to location as either urban/semi-urban or remote/island, and the former showed higher scale efficiency (91.9% vs. 75.3%, Mann-Whitney *P *< 0.001), while the latter better technical efficiency (81.1% vs. 75.7%, Mann-Whitney *P *= 0.103).

**Table 2:** CRS, VRS and scale efficiency overall and by unit type, size and location

	**CRS efficiency**	**VRS efficiency**	**Scale efficiency**
**Overall **(N = 194)	67.28	77.05	87.68
**By type**			
NHS (N = 103)	59.59	70.10	85.86
IKA (N = 91)	75.99	84.90	89.74
*P-sig.*^1^	*<0.001*	*<0.001*	*0.080*
**By size**			
Small (N = 66)	63.81	84.15	75.90
Medium (N = 60)	66.39	72.35	90.87
Large (N = 68)	71.44	74.29	96.31
*P-sig.*^2^	*0.126*	*0.002*	*<0.001*
**By location**			
Urban/semi urban (N = 145)	69.48	75.68	91.87
Remote/island (N = 49)	60.77	81.10	75.28
*P-sig.*^1^	*<0.05*	*0.103*	*<0.001*

^1^According to Mann-Whitney test.

^2^According to Kruskal-Wallis test. Note: Size designated according to catchment population, i.e. small (<15,000), medium (15,000–30,000) and large (>30,000)

VRS efficiency was further analyzed and the results are presented in Table 3, in the form of efficiency statistics and rankings by primary care provider and unit size. Evidently, IKA facilities were more technically efficient than NHS ones, in all size categories, with profound differences in medium and large units. Larger NHS centers appeared to suffer from an average technical inefficiency of approximately 36.5%. As for identified benchmarks (i.e. 100% efficient units), large NHS centers were those with the fewest (3 compared to 12 from IKA) and overall more benchmarks were identified in IKA facilities compared to the respective NHS ones. These findings could perhaps be regarded as warning signals for NHS facilities to attempt to emulate the production practices of their IKA counterparts. However it is questionable if this is feasible, or even plausible. It should be noted that a problem with DEA, when specifying VRS, is that units at both ends of the size classification may be identified as efficient simply because of a lack of other comparable units [37]. This probably explains why among the smallest and largest health centers, the proportion of technically efficient units was higher than among medium-sized units, a finding which has also been reported elsewhere [12].

**Table 3:** VRS technical efficiency statistics and rankings

	**NHS primary care centers**			**IKA primary care centers**		
		
**Statistics**	**Small (N = 36)**	**Medium (N = 35)**	**Large (N = 32)**	**Small (N = 30)**	**Medium (N = 25)**	**Large (N = 36)**
		
Mean	80.77	65.20	63.47	88.21	82.35	83.91
Median	85.87	64.08	61.07	97.33	87.67	85.76
Minimum	28.89	31.97	25.58	59.29	45.12	50.30
Maximum	100.00	100.00	100.00	100.00	100.00	100.00
**Rankings**						
						
100%	9 (25.0%)	3 (8.6%)	3 (9.4%)	14 (46.7%)	7 (28.0%)	12 (33.3%)
80–99.9%	11 (30.6%)	5 (14.3%)	7 (21.8%)	7 (23.3%)	8 (32.0%)	11 (30.6%)
60–79.9%	10 (27.7%)	13 (37.1%)	6 (18.7%)	7 (23.3%)	6 (24.0%)	8 (22.2%)
40–59.9%	4 (11.1%)	9 (25.7)	10 (31.4%)	2 (6.7%)	4 (16.0%)	5 (13.9%)
< 40%	2 (5.6%)	5 (14.3%)	6 (18.7%)	0 (0.0%)	0 (0.0%)	0 (0.0%)

Note: Size designated according to catchment population, i.e. small (<15,000), medium (15,000–30,000) and large (>30,000)

Table 4 presents scale efficiency statistics by provider and facility size. Medium- and large-sized IKA facilities and large NHS facilities performed better in respect to scale of production. On the other hand, smaller centers were more scale inefficient, particularly those operating under the NHS (74.3%). Approximately 3/4 of the entire sample (145 out of 194) operated under increasing returns to scale (IRS), implying that these units theoretically should attempt to increase efficiency by scaling their production upwards. By provider, 83.5% (86 out of 103) of NHS and 64.8% (59 out of 91) of IKA facilities operated under IRS. Contrarily, the 28 facilities (10 NHS and 18 IKA) operating under decreasing returns to scale (DRS) serve mostly large populations and ideally should scale down their production to improve efficiency. Only 21 facilities were scale efficient (7 NHS and 14 IKA) with an average catchment population of 35,000.

**Table 4:** Scale efficiency statistics and returns to scale

	**NHS primary care centers**			**IKA primary care centers**		
		
**Statistics**	**Small (N = 36)**	**Medium (N = 35)**	**Large (N = 32)**	**Small (N = 30)**	**Medium (N = 25)**	**Large (N = 36)**
		
Mean	74.33	87.53	97.01	77.77	95.55	95.68
Median	80.36	91.09	98.52	77.99	98.59	99.43
Minimum	27.18	45.56	83.65	44.10	75.61	74.63
Maximum	100.00	100.00	100.00	98.64	100.00	100.00

**Returns to Scale (% N)**						
						
Increasing (IRS)	32 (88.9%)	32 (91.4%)	22 (68.7%)	30 (100.0%)	17 (68.0%)	12 (33.3%)
Constant (CRS)	3 (8.3%)	2 (5.7%)	2 (6.3%)	0 (0.0%)	6 (24.0%)	8 (22.2%)
Decreasing (DRS)	1 (2.8%)	1 (2.9%)	8 (25.0%)	0 (0.0%)	2 (8.0%)	16 (44.4%)

Note: Size designated according to catchment population, i.e. small (<15,000), medium (15,000–30,000) and large (>30,000)

**Figure 1:**
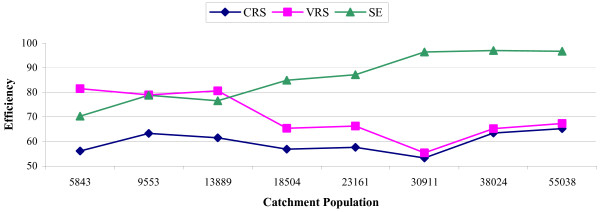
CRS, VRS and scale efficiency by catchment population for NHS primary care centers.

**Figure 2:**
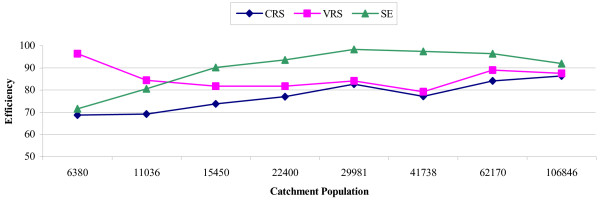
CRS, VRS and scale efficiency by catchment population for IKA primary care centers.

Figures 1, 2 further elucidate the effect of catchment population on CRS, VRS and scale efficiency by depicting efficiency alterations with increasing population, for the NHS and IKA respectively. Specifically, figure 1 (NHS) shows opposite trends for technical and scale efficiency, which became more obvious in populations of approximately 18,000 and thereafter appeared to differ increasingly as population size increased. Simply put, the larger the catchment population (i.e. semi-urban and urban areas), the greater the variation between technical and scale efficiency, implying that for these larger areas, policymakers have more clues on where attention is required. The same generally held for IKA facilites (Figure 2), however the opposing trends emerged in smaller populations (of approximately 12,000) and started to converge in large ones (>100,000).

It is a social policy of the NHS to provide health care coverage to remote rural areas and distant islands, at the expense of efficiency, in an attempt to help these areas maintain their populations. In order to disentangle the effect of the geographic factor on the efficiency of urban and semi-urban facilities, further analysis was performed and the results are shown in Table 5. Specifically CRS, VRS and scale efficiency from DEA analyses with all facilities (N = 194) are presented by location and provider. Apparently, IKA performed better regarding technical efficiency in both urban/semi-urban and remote/island areas, whereas the two providers were fairly equivalent in terms of scale efficiency. A second, more homogenous and focused DEA was performed with a new sample containing only urban/semi-urban NHS (N = 67) and IKA (N = 78) units. The results (Table 5) showed that there was indeed a scale efficiency difference, in favour of IKA, which was apparently masked, in the previous analysis, by the presence of the remote/island facilities, (90.8% vs. 86.7%, Mann-Whitney *P *< 0.01). As for technical efficiency, the scores remained significantly higher for IKA.

**Table 5:** CRS, VRS and scale efficiency by unit location and type

**Facility location**	**CRS efficiency**	**VRS efficiency**	**Scale efficiency**
Urban/Semi urban (N = 145)			
NHS (N = 67)	60.74	66.31	91.80
IKA (N = 78)	76.99	83.72	91.94
*P-sig.*^1^	*<0.001*	*<0.001*	*0.380*
Remote/Island (N = 49)			
NHS (N = 36)	57.44	77.15	74.82
IKA (N = 13)	69.99	92.01	76.57
*P-sig.*^1^	*0.102*	*<0.01*	*0.928*
**Urban/Semi urban centers**^3^			
			
NHS (N = 67)	61.13	70.34	86.69
IKA (N = 78)	77.02	84.83	90.77
*P-sig.*^1^	*<0.001*	*<0.001*	*<0.01*

^1 ^According to Mann-Whitney test.

^2 ^Remote and island centers have been excluded from the DEA analyses

The next step in the analysis was to determine efficiency targets, which in an input-oriented approach correspond to the (theoretically) required mean input reduction in order for units to become efficient. These results, by facility type, size and location are presented in Table 6. In the NHS, there appeared to be a general overabundance of administrative and other support staff in all three size categories and in both urban/semi-urban and remote/island regions. The same can be said for nursing/paramedical staff, particularly in medium- and large-sized NHS facilities. In the case of IKA, the three types of personnel apparently affected efficiency equally. It should be noted that although efficiency targets are theoretically feasible, staff reductions in the Greek NHS are practically impossible due to various social, political and other factors.

**Table 6:** Mean efficiency targets^1 ^by facility type, size and location

		**Inputs**		
		
**Facilities (N)**	**N (%) inefficient**^2^	Physicians	Nursing/Paramedical staff	Administrative/other staff
**NHS centers**				
Small (36)	27 (75.0%)	25.6%	26.4%	46.4%
Medium (35)	32 (91.4%)	38.1%	40.6%	54.6%
Large (32)	29 (90.6%)	40.3%	48.2%	51.4%
				
Urban/semi urban (67)	60 (89.6%)	37.6%	42.7%	52.6%
Remote/island (36)	28 (77.8%)	29.4%	30.4%	47.7%
				
**IKA centers**				
Small (30)	16 (53.3%)	22.3%	22.1%	22.9%
Medium (25)	18 (72.0%)	25.0%	27.0%	24.5%
Large (36)	24 (66.7%)	27.6%	26.2%	25.8%
				
Urban/semi urban (78)	54 (69.2%)	25.3%	25.2%	24.5%
Remote/island (13)	4 (69.2%)	26.0%	26.0%	26.0%

^1 ^Refers to the required reduction (%) in input in order to achieve 100% efficiency.

^2 ^Refers to the total number (%) of <100% efficient facilities in each category

In the second stage of the analysis, multivariate Tobit models were formulated in order to determine the effect of non-discretionary inputs on technical and scale efficiency. The results are presented in Table 7. Obviously both models, as a whole, were statistically significant (Chi-square P < 0.001). Pseudo R^2 ^values were 0.031 and 0.053 respectively. However, this may not be the best measure of fit and, hence, was improved by calculating R^2 ^between predicted and observed values. The R^2 ^values were now 0.205 and 0.348 respectively, which are much closer to what an OLS regression would have given. Systematically lower technical efficiency scores corresponded to the NHS, to medium- and large-sized centers, and to urban/semi-urban areas. Two exogenous factors, namely unit type and size, were significant predictors of technical efficiency, whereas location was at the borderline (*P *= 0.061). In the case of scale efficiency, lower scores appeared in remote/island locations and in smaller facilities, whereas facility type was evidently not a significant predictor (*P *= 0.218), in accordance to the results presented in Table 2 as well.

**Table 7:** Tobit regression analyses

	MODEL_1 – Technical efficiency (VRS)				MODEL_2 – Scale efficiency			
		
**Explanatory Variables**	Coefficient	Std. Err.	*t*-ratio	*P*-value	Coefficient	Std. Err.	*t*-ratio	*P*-value
	
Constant	75.93	4.36	17.43	0.000	79.58	2.57	30.94	0.000
TYPE_2	20.90	3.62	5.77	0.000	2.62	2.11	1.23	0.218
SIZE_2	-11.59	4.58	-2.53	0.012	12.91	2.71	4.77	0.000
SIZE_3	-10.25	4.64	-2.21	0.028	17.62	2.74	6.42	0.000
LOCAT_2	8.79	4.67	1.88	0.061	-8.75	2.70	-3.24	0.001
Log likelihood	-708.701				-716.349			
Chi-square	44.33 *				80.57 *			
Pseudo R^2^	0.031				0.053			

TYPE_2: IKA units, SIZE_2: medium size units, SIZE_3: large size units, LOCAT_2: remote/island units

*P < 0.001

## Discussion

This study compared technical and scale efficiency of primary care centers from the two largest Greek providers, the NHS and the Social Security Foundation (IKA) and investigated how each type of efficiency was affected by the size of the facilities-proxied by the size of the eligible service population- and by geographical position. The results revealed variations between and within the providers. We suggest that efficiency in primary care provision in Greece can be improved through better resource management. To achieve this, policymakers need information regarding relative performance of providers and facilities, in order to plan a strategy for optimal service provision and to overcome opposition to change posed by various special interest groups [38].

Under the CRS assumption, facilities suffered from similar degrees of inefficiency, regardless of size. After decomposing into VRS technical efficiency and scale efficiency, clear trends emerged, implying that both types of efficiency should be estimated before conclusions are drawn. Smaller facilities (catchment population < 15,000) were observed to suffer mostly from scale inefficiencies and, theoretically, they should attempt to scale up production in order to improve efficiency. However this is not really controllable due to the fact that small centers are mainly located on islands or in various remote rural areas. Conversely, larger primary care centers (catchment population > 30,000) located mainly in suburban areas closer to hospitals, evidently suffered more from technical inefficiency. The latter is dependent on the input-output mix which is theoretically controllable, but in practice is substantially limited by the general preference of health care staff-mostly physicians- to work in these facilities (and not in the smaller ones) for reasons such as professional development and career advancement, since they are closer to urban areas.

IKA primary care centers exhibited 7.4% to 20.4% (by catchment population) higher technical efficiency than NHS health centers, and 3.4% to 8.0% higher scale efficiency (in small- and medium-sized facilities respectively). It would be misleading and perhaps unfair to indisputably accept that IKA is more efficient than the NHS in the primary care arena, without assessing other existing information and contributing factors. One such example is differences in the populations addressed by the NHS and IKA facilities (rural versus urban). People living in, or close to, major urban areas have easier access to the respective primary care facilities. They are also more health conscientious, have a different epidemiological profile and in conjunction with various influencing sociodemographic factors such as age and education, they make greater utilization of health services [39].

The two Greek providers are organized and function in different ways and this also affects efficiency. NHS primary care physicians are often committed by informal interpersonal relationships with patients of the local rural community, particularly the elderly and chronically ill, and frequently make time-consuming house visits. Furthermore, NHS facilities operate on a 24-hour basis and often deal with difficult emergency cases in need of specialized medical attention. Most of these cases are referred to secondary or tertiary regional hospitals, and the health centers usually support the transport with medical and other personnel. IKA health centers, on the other hand, operate during "office hours" and handle only scheduled patient visits.

Another point worth noting is that the NHS centers often support affiliated rural posts with staff, which is not relieved of other daily responsibilities at the health centers. Furthermore, staff synthesis itself is quite different between the NHS and IKA. In the former, many non-medical specialties are employed, e.g. social workers, gardeners, drivers, etc. and these employees are not directly involved in the production process. However, they are regarded as inputs as far as DEA is concerned. Contrarily, IKA health centers operate primarily with medical staff of differing specialties and nursing/paramedical staff.

Both the NHS and IKA have envisaged activities in the fields of preventive medicine, health promotion and health education, by addressing hygiene, (e.g. dental hygiene education at schools), population screening, vaccinations, pap-tests, prenatal control, family planning, blood donation and other services intended to promptly diagnose symptoms and protect the population from serious diseases. Although both sectors have failed to follow through on these intensions, it is clear that the NHS centers, particularly the more remote ones, have done much more in this area than their urban IKA counterparts [31]. However, these services were not reflected in the DEA outputs used in this study and this creates an unfavourable situation, regarding efficency, for the NHS primary care centers.

An effective primary care system is associated with increased health for the population, which in turn means less consumption of expensive diagnostic and therapeutic medical interventions. In health economics terms this facilitates generation of funds to be allocated elsewhere, to serve other needs within the health system [40]. Whilst efficiency is a predominant criterion for resource allocation, typically it is not the only one favoured by society and its elected or appointed agents (i.e. politicians and/or policy-makers). The other equally important criterion is equity and it is taken as axiomatic that health care pursues to improve health and to reduce access inequalities, and that policymakers seek to concurrently achieve the, somewhat contradictive, goals of efficiency and equity [41,42].

The results of this study raise the question if the theoretically possible efficiency improvements are actually plausible and desirable. Assuming that the number of patients is uncontrollable, efficiency improvements imply the reduction of resources. From a social perspective, this is undesirable for populations with already limited health care options. Approximately 35% of the NHS primary care centers in this sample are located in remote regions or on small islands, which are often occluded from the mainland, particularly in the winter. It should be also noted that most employees in these facilities originate from close-by regions and desire to work in, or close to, their hometowns. It would be perhaps contradictory to the equity criterion to limit staff, despite our results suggesting otherwise. Achieving efficiency targets requires reducing nursing/paramedical and administrative/other staff in small and remote facilities. Possible solutions could be for management to attempt redistributing personnel within each unit and exploring the possibility of setting staff performance incentives.

To account for the NHS's social policy to maintain facilities in small and remote areas, a second and more focused DEA was performed including only NHS and IKA facilities located in urban or semi-urban regions. This resulted in a 5.1% reduction of mean scale efficiency in NHS facilities, which was now statistically significantly different (and lower) from the mean scale efficiency of the IKA centers. This implies that the relatively low scale efficiency of small and remote facilities increased the mean score of the entire sample in the previous analysis, since larger units were assigned higher scores (because DEA compares units in relation to peers). On the other hand, this second analysis did not produce different results in respect to technical efficiency.

This study has some limitations as well. Staff is generally an unstable variable, with wide variability in levels (staff skill mix) and ratios across settings and constitutes a potential source of efficiency differences. This may have introduced ambiguity in the results since significant staffing inequalities were observed, mostly at the expense of remote and island facilities because it is difficult to recruit physicians in sufficient numbers due to the living conditions in these particular regions of the country. Moreover, medical specialists feel occluded from the "technologically dynamic" urban environment as well as from their professional colleagues. The result is that the best-staffed facilities are mostly those close to major urban areas.

Catchment population is also somewhat vague and difficult to measure precisely, as population varies between summer and winter (e.g. on islands) or because patients often prefer centers more easily accessible rather than those to which they are theoretically assigned based on geographical criteria (e.g. people living close to the border between two prefectures). In the case of facilities close to highly populated urban areas, patients often prefer to directly visit hospital outpatient departments. The borderline between primary and secondary care in Greece is often vague. Due to the absence of a referral system, patients are practically free to refer themselves to any service provider and this is mostly responsible for the significant use of hospital outpatient departments as a first point of contact [2,26].

A final point requiring attention is that efficient facilities are not necessarily producing high quality outputs. In the present study however, quality of outputs was assumed to be fairly similar in all units. This is justified by a number of reasons: i) many patients (e.g. the chronically ill) visit the centers repeatedly for prescriptions, ii) physician-patient contact is usually brief regardless of case, iii) physicians typically refer "difficult" cases to secondary hospitals and iv) a significant number of patients visit the centers for multiple re-examinations. In any case, it has been shown that the inclusion of quality measures highlights weaknesses with basic DEA models [43].

## Conclusion

In the present study, smaller primary care centers appeared to suffer mostly from scale inefficiencies and larger ones from technical inefficiencies. IKA facilities evidently outperformed NHS ones, irrespective of population size and location, but this should be interpreted in light of various functional, organizational and environmental differences between the two major providers. NHS facilities showed higher technical and scale efficiency variations between small and large catchment populations. The ideal service population, from the efficiency point of view, was approximately 35,000. Below and above this threshold, IRS and DRS scale inefficiencies were observed respectively. However, it must be emphasized that population thresholds cannot be generalized and more research is required in this direction. In light of the current government's will to set up NHS urban health centers and expressed proposals for an integrated primary care system in Greece [44], efficiency studies can offer valuable insight, particularly regarding staffing policies.

## Authors' contributions

**NK **analyzed and interpreted the data and drafted the manuscript. **GM **collected the data and assisted in the statistical analyses. **VHA **participated in the design of the study and revised the manuscript for intellectual content. **DN **conceived the study and participated in its coordination. All authors have read and approved the final manuscript.
